# Radiation therapy in functioning and no functioning pituitary neuroendocrine tumor: systematic review of the recent literature after 2011

**DOI:** 10.3389/fendo.2024.1468724

**Published:** 2024-11-12

**Authors:** Racha-Miloda Hemaidia, Hélène Cebula, Bernard Goichot, Georges Noel

**Affiliations:** ^1^ Radiotherapy Department, Institut de Cancérologie StrasTbourg Europe (ICANS), Strasbourg, France; ^2^ Neurosurgery Department, Hautepierre University Hospital, Strasbourg, France; ^3^ Endocrinology Department, Hautepierre University Hospital, Strasbourg, France

**Keywords:** pituitary, adenoma, PiNET, radiotherapy highlights, radiation therapy

## Abstract

Neuroendocrine pituitary tumor, a benign cells proliferation, can cause significant morbidity due to its local invasiveness and secretory properties. Historically, radiotherapy has been employed as a second or third-line treatment option, with studies dating back to the mid-20th century. However, advancements in radiotherapy techniques, such as intensity-modulated radiation therapy (IMRT), stereotactic radiosurgery, and proton therapy, have revolutionized treatment approaches. This review aims to critically evaluate the recent literature (2011–2022) on the use of radiotherapy in both functioning and nonfunctioning neuroendocrine pituitary tumor. We employed the PRISMA (Preferred Reporting Items for Systematic Reviews and Meta-Analyses) methodology to systematically analyze 52 articles, focusing on local and hormonal control, radiotherapy protocols, and treatment-related side effects.

## Highlights

- Radiation Therapy Effectiveness: Retrospective studies indicate radiation therapy’s effectiveness, showing good local response rates and acceptable hormonal control.
- MRI Importance: High-quality brain MRI with contrast-enhanced T1-weighted and T2 fat-saturated thin-slice sequences is vital for precise treatment planning.
- Predictive Factors: Response to radiotherapy depends on factors like margin and maximum dose, tumor volume, extension, and initial hormonal levels.
- Main Side Effects: The primary side effect is hypopituitarism, entwined with preexisting conditions and the tumor’s hormonal repercussions. Other adverse effects are rare, and the benefits, particularly regarding visual outcomes, outweigh the risks.

## Introduction

Pituitary neuroendocrine tumors (PiNETs), according to the new WHO 2022 classification and previously referred to as pituitary adenomas, represent a proliferation of pituitary cells that is considered benign but has the potential for local invasion and aggressiveness (1). The prevalence of these tumors has increased due to the widespread utilization of magnetic resonance imaging (MRI) for various indications as headaches, seizures, and visual deficits. Unlike computed tomography (CT), MRI has the capability to detect small PiNETs that would otherwise remain invisible (2). According to the largest brain tumor registry in the United States, the incidence rate stands at 4.07 cases per 100,000 individuals per year, with no significant gender-based disparities. The prevalence of different subtypes of PiNETs is as follows: clinically functioning lactotroph tumors (66.2%), clinically non-functioning pituitary tumor (NFPT) (14.7%), clinically functioning somatotroph tumors causing acromegaly (13.2%), clinically functioning corticotroph tumors causing Cushing’s disease (5.9%), and the remaining 1% comprises other types such as thyrotroph tumors (TSH), gonadotroph tumors, and more (3).

Despite their benign nature, PiNETs have the potential to compress or invade surrounding tissues and organs, including the optic pathway, brainstem, and cavernous sinus. Furthermore, these tumors can secrete hormones, leading to disturbances in the hormonal system and consequential diseases with significant prognostic implications (4).

The primary treatment options commonly recommended for patients with these pathologies include surgical intervention and medical treatments (5). Radiotherapy has been recognized as an effective treatment since the 1960s (6, 7). However, its use is generally limited to the second or third line of treatment, potentially due to the side effects that were associated with older techniques. Additionally, concerns about radiation-induced cancers persist, particularly among younger patients who have several decades of life ahead of them (8). Over the past two decades, advancements in stereotactic irradiation and intensity-modulated radiotherapy (IMRT) have significantly improved the protection of critical organs surrounding the pituitary gland (9, 10). The aim of this review is to analyze the results of the most recent publications using the IMRT or stereotactic irradiation on local and hormonal control and its side-effects.

## Method

The PRISMA (Preferred Reporting Items for Systematic Reviews and Meta-Analyses) method was employed in this study (11). A comprehensive search of articles was conducted in the Medline database using specific keywords related to PiNETs, including “pituitary adenoma,” “Cushing,” “Acromegaly,” “prolactinoma,” “TSH-secreting,” “Nelson syndrome,” combined with “radiation therapy” or “radiotherapy.” The search was limited to articles published from 2011 onwards. Approximately 1500 publications were initially identified.

The selection process for the final articles involved two stages: title screening and abstract review. Inclusion criteria were applied, which included articles written in either French or English, studies involving more than 20 patients, reports describing the technique of radiotherapy, classification of PiNETs subtypes, and providing details on local control or hormonal control outcomes, as well as side effects.

## Results

**Figure 1:**
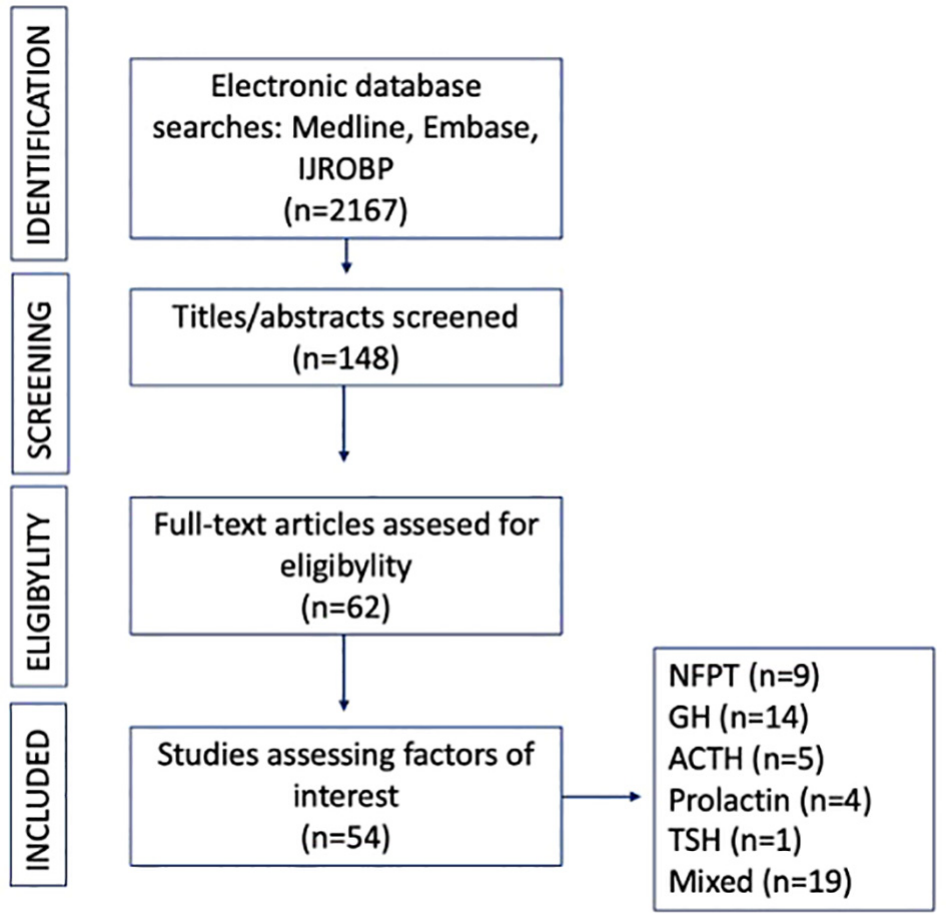
Flow-chart: Nonfunctioning pituitary tumor (NFPT), growth hormone (GH), adrenocorticotropic hormone (ACTH), Thyroid stimulating hormone (TSH).

From 2011 to 2021, 52 articles were selected for analysis (**Figure 1**). All the included studies were retrospective (**Tables 1**-**6**). Among them, thirteen studies provided data on patients from multiple centers. The number of patients enrolled in the studies varied, ranging from 21 to 1023. Treatment periods spanned from 1964 to 2019. Median follow-up ranged from 33 to 198 months (**Tables 1**–**6**).

**Table 1:** NFPT studies.

Author	Date	Patients	MRI	Tumor volume	Technique, dose, fractions	Local control	Predictive factor	Side effects
Deng et al. (27)	2019	90	—	—	- >3 cm: NFRT; 46-50Gy - < 3 cm: FSRT; 21 Gy/3fr or 12-14/1fr Gy	92.2% (vs 75.8% if no RT) *(p=0.009)*	-PTV>3 cm -cavernous sinus invasion	- hypopituitarism: 23.1% vs 25% if no RT - oculomotor paralysis: 3.85%
Gopalan et al. (24)	2011	48	- 1.2mm slice - T1/fat sat gadolinium	—	GK, SRS: 18.4 Gy (8-25 Gy)	83%	- volume>5ml - margin dose<12Gy	- visual deficit: 2% - hypopituitarism: 39%
Hata et al. (72)	2021	32	- T1 gadolinium	—	FSRT: 31.3 Gy (17.2-39.6)/8 fr (6–15)	97%.	—	- hypopituitarism: 3% - visual deficit: 6%
Itawa et al. (14)	2011	100	- 1.5 T - 2 mm slice - T1 gadolinium	5.1 (0.7-64,3)	FSRT: - 17-21Gy/3 fr (83%) - 22-25Gy/5 fr (17%)	97%	—	- visual disorder: 1% grade 2 - hypopituitarism: 3%
Oh et al. (47)	2018	76	- T1/T2 - gadolinium	—	GK, SRS: 20.6+/-0.7 Gy	96%	—	- Hypopituitarism: 24.5% (GH=33%, TSH= 22%, ACTH=19%, prolactin= 15%) - PF: tumor volume, distance between gland and tumor, stalk dose, normal gland dose
Sadik et al (28)	2017	50	- Axial: 1.5mm slice - Coronal: 0.8mm slice - T1 gadolinium	3.4 (0.2-11.1)	GK, SRS 14Gy (6.9-33.3Gy)	96%	volume >3.4cm3	hypopituitarism: 22% (RT adjuvant 4% vs delayed 18%)
Wilson et al. (50)	2012	217	Yes	- SRS:2.4 (0,3–9) - FSRT: 6.8 (0.2-115.6)	- SRS: 14Gy (12–25) - FSRT: 50Gy (14.4-53.6)/28 fr - NFRT: 50.4 Gy (30–76)/28 fr	- SRS: 88%; - FSRT 88%	—	- hypopituitarism: SRS 10%, FSRT 7%; - Visual: SRS 0%; FSRT 2%; CRT 11%; - Memory: SRS1%, CRT 4%; - Epilepsy: SRS2%, FSRT 2%, CRT 6%
Yu et al. (32)	2020	81	- Thin slice - T1 - gadolinium	2.3 (0.1-31.3)	GK, SRS 13Gy (8-22Gy)	88.9%	- Volume>3cm3 - Margin dose <12Gy	- Vision 5%, - hypopituitarism 17.3% (MT: 73 months)

NFPT (Nonfunctioning pituitary tumor), FPT (functioning pituitary tumor), SRS (stereotactic radiosurgery), FSRT (fractionated stereotactic radiosurgery), NFRT (normo fractionated radiotherapy).MT (median time), fr (fractions), PF (predictive factor).

**Table 2:** Somatotroph clinically functioning pituitary tumors studies.

Author	Date	Patient	MRI	Volume (cm^3^)	Dose	LC	HC	Predictive factors	Side effects
Alonso et al. (74)	2019	21	—	—	GK, SRS: 17Gy (9–35), or 23Gy (12–35)	83.3%	42%	—	-Visual: 10% -Hypopituitarism:19%
Balossier et al. (33)	2020	42	- 1mm slice - T1/T2/fat sat, - gadolinium	0.9 (0.13-8)	SRS: 28 Gy (20–35)	100%	-HN: 72.4% -WM: 52%	IGF1 pretherapeutic level	-hypopituitarism: 19.3%; -visual deficit improvement: 2.6%(RT) vs 4.8% (No RT)
Bostrom et al. (15)	2014	35	-1.5T -0.7-1.2mm -T1 gadolinium	—	-SRS <4cm: 20Gy; (12 patients) -FSRT> 4cm: 1.8-2Gyx25-30	97.1%	-HN: 23%	SRS	-Hypopituitarism: 46% -Visual field: 2.85%, -oculomotor: 2.85%
Crouzeix et al. (51)	2018	46			45-50 Gy/25	95.9%			QoL significantly worse in RT groupcogn
Ding et al. (35)	2019	371	—	2.5 ± 2.9	GK, SRS: 24.2 ± 6.4 Gy	98.8%	-Medication lowering: 56% (MT: 38 months)	Initial level of IGF1	-hypopituitarism: 26% -neuropathy II: 3.5%, -cranial neuropathy: 4.3%,
Gonzales Virla et al. (36)	2019	94	—	—	-NFRT: 52 Gy/26fr	—–	-HN: GH 41%, IGF1 = 50.8%	pre radiotherapy IGF1 level	-hypopituitarism: FSH: 20%; Cortisol: 18%; TSH:29% Neuropathy: 1% -Meningioma 1%
Knappe et al. (75)	2020	352	—	—	SRS vs FSRT	—	-FSRT=48%; -SRS=52% (p=0.74)	—	0R=0.54 (0.30–1.00, P=0.049) for SRS compared to FRT
Kong et al. (38)	2018	138	-1.5mm slice -T1/T2, gadolinium	1.0 (0,1–10,3)	SRS: 25 Gy (12-35Gy)	—	-HN: 58% (MT: 138 months)	CH: female patient (p=0.004); IGF1level; GKS as an adjuvant treatment (p=0.001; p=0.01)	-hypopituitarism: 8.6%
Lee et al. (20)	2015	73	yes	2.8 (0, 3–13)	SRS: 25 Gy (9–30 Gy)	97,3%	75.3% (MT: 26 months)	margin dose maximum dose pre-SRS IGF-1 level	hypopituitarism: 38%
Pai et al. (40)	2018	76	yes	2.8 (1.4-5.6)	SRS: 15.8Gy (12.5-18)	98%	43,3%	Cavernous invasion, IGF-1 level	hypopituitarism: 11.8%
Patibandla et al. (22)	2018	157	-1mm slice, -Axial, coronal -T1/Fat Sat gadolinium	2.4+/-3.1	SRS: 22.8+/-2 Gy	—	64.9%	—	hypopituitarism: 32.5%
Patt et al. (12)	2015	36	yes		NFRT: 45Gy/25 fractions	100%	55%	—	hypopituitarism: 33%
Sims-William et al. (53)	2021	104	-T1 gadolinium	2.0	SRS and FSRT: 30Gy (17.2Gy-38Gy)	—	92%	—	-hypopituitarism: 38% -visual: 5.7% -ophthalmoplegia:6,7% -stroke: 2.9% -trigeminal neuralgia: 1%
Wu et al. (42)	2021	75 (37 A vs 38 1^st^)	-1mm slice	1^st^ =1,0 (0.6-1.8) A=1.6 (1,2–2,5)	SRS: -A: 25Gy (21–28) -1^st^: 28Gy (26–30)	Regression: 1^st^ =66,6% vs A=74.07% (p=0.56) Stable 1^st^=25.9% vs A=14.82% (p=0.3); Progression: 1st=7.4% vs A=11% (p=0.97)	-1^st^ =23,68% -A=27% (p=0.94)	nadir GH after RT;	-hypopituitarism: 1st=10.53% mean time 10.75 months; A=21.62% time 24.13 months (p=0.1 -Visual: 1st=7,8%; A=5,41% (p=1)
Lian et al. (13)	2020	113	-2-3mm	—	NFRT: -CTV: 50-56 Gy 25-30fr -GTV(SIB): 60.2 Gy/28 fr	99%	74.3% (MT: 36.2 months)	>33years tumor size	-hypopituitarism: 28.3% -stroke: 0.9%

LC (local control), HC (Hormonal control), NFPT (Nonfunctioning pituitary tumor), FPT (functioning pituitary tumor), SRS (stereotactic radiosurgery), FSRT (fractionated stereotactic radiosurgery), NFRT (normo fractionated radiotherapy), **MT** (median time); HC (hormonal control), A (adjuvant), 1^st^ (First line treatment), fr (fractions), HN (hormonal normalization), WM (hormonal normalization without medication)

**Table 3:** Corticotroph clinically functioning pituitary tumors studies.

Author	Date	Patient	MRI	Volume (cm^3)^	Dose	LC	HC	Predictive factors	Side effects
Apaydin et al. (52)	2020	38	—	—	Mean: 25.9 Gy +- 3.6	95%	52%; (MT: 15 months)	–	-hypopituitarism: 31% (MT: 10.5 months) -stroke: 10.5% (MT: 31 months)
Balossier et al. (71)	2021	26	-1mm slice -T1/T2/fat sat	—	28.5Gy (24-35	100	69.2% at 36 months	—	hypopituitarism: 32% at 3 years
Marek et al. (76)	2014	26	-T1/T2	2,3	30 Gy (19-35	91%	80.7% (MT: 30 months)	–	hypopituitarism: 23%
Mehta et al. (30)	2017	278	yes	1.7 (0,01-12.4)	23.7+/-6.2 Gy (3–40)	95%	80% (MT: 14.5 months)	margin and maximum dose	-Hypopituitarism: 25% -visual: 1%, -cranial neuropathy: 1%
Shepard et al. (18)	2018	346	yes	2.6 +/- 2.3	22.4Gy+/-6.2 (3–35)	92.7%	-HN:79.4% -WM: 63.2%	Volume >1.6 cm3 no prior radiotherapy margin dose maximum dose	-Hypopituitarism: 22.7% -Vision: 2.9% -cranial nerve deficit: 2.9%

LC (local control), HC (Hormonal control) NFPT (Nonfunctioning pituitary tumor), FPT (functioning pituitary tumor), SRS (stereotactic radiosurgery), FSRT (fractionated stereotactic radiosurgery), NFRT (normo fractionated radiotherapy), **MT** (median time); HN (hormonal normalization), WM (hormonal normalization without medication).

**Table 4:** Lactotroph clinically functioning pituitary tumors studies.

Author	Date	Patient	MRI	volume	Dose	LC	HC	Predictive factors	Side effects
Cohen Inbar et al. (25)	2015	38	-1.3-3mm slice -Fat Sat, gadolinium	2.42 (0.1–6.4)	25Gy (5.2–25)	92,1%	-WM: 50% (MT: 20 months)	Cavernous sinus invasion	hypopituitarism: 26.3%
Hung et al. (37)	2019	289	—	—	SRS	95%	-WM at 8 years: 54%	Pretreatment level	-hypopituitarism:25% -Visual:3%
Jezkova et al. (77)	2019	28	-1.5T -2mm slice -T1/T2 gadolinium	1,2 (0.8-13.2)	35 Gy (20-36Gy	100%	-HN: 82.1%; -WM: 46.4% (MT: 152 months)	—	-Hypopituitarism: 8.3% -Visual field: 3,5%
Li et al. (78)	2020	24	gadolinium	—	15 Gy (10.5 to 23.6)	100%	-HN: 66.7%: -WM: 41.7%,	—	-hypopituitarism 16.7% (FSH 12.5%; TSH 4.2%)

LC (local control), HC (Hormonal control), NFPT (Nonfunctioning pituitary tumor), FPT (functioning pituitary tumor), SRS (stereotactic radiosurgery), FSRT (fractionated stereotactic radiosurgery), NFRT (normo fractionated radiotherapy), **MT** (median time); HN (hormonal normalization), WM (hormonal normalization without medication).

**Table 5:** Thyrotroph clinically functioning pituitary tumors study.

Authors	date	Patients	MRI	volume	Dose	LC	HC	Predictive factors	Side effects
Malchiodi (79)	2014	70	—	—	-NFRT (32%): 46-54Gy -SRS (68%): 12-25Gy	78%	-WM: 63%	—	hypopituitarism: 32%

LC (local control), HC (Hormonal control), NFRT (normo fractionated radiotherapy), SRS (Stereotactic radiosurgery), WM (hormonal normalization without medication).

**Table 6:** Studies including multiple types of PiNETs, NFPT and FPT.

Authors	Date	Patients	MRI	Volume	Dose	LC	HC	Predictive factors	Side effects
Albano et al. (16)	2018	47	Yes	3.9 (0.3-16.3)	-NFPT: 21Gy/3fr -FPT: 30Gy/3 fr	100%	—	—	hypopituitarism: 25%
Barber et al. (69)	2015	75	-1.5mm slice -T1/Fat Sat gadolinium	19+/-11.3 (2.5-49.9)	49.28 +/-6.5Gy	100%	-WM: 53.8% (MT: 26 month)	—	-Hypopituitarism: 28% -Vision: 1.5%;
Cohen Inbar et al. (70)	2016	60	Fat Sat	1.3 (0.3-13.4)	25Gy (6–30)	93.3%	—	—	hypopituitarism: 11.7%
Cordeiro et al. (26)	2019	1023	-1mm slice -Fat Sat gadolinium	—	-FPT: 25+/- 6.5 Gy; -NFPT: 16+/- 4.23 Gy	96,3%	—	margin dose maximum dose supra-sellar extension treatment volume	Hypopituitarism: 24.2%
Gupta et al. (19)	2018	46	-Thin slice -gadolinium	—	25 Gy (12–40)	100%	51%; at 5 years: -Acromegaly=28% -Cushing=81%	larger PTV	-Hypopituitarism: 19.6% -Diplopia: 2%
Kim et al. (17)	2013	76	-1.5T -0.9mm slice -gadolinium	5.3 (0,3-16.5)	50.4 Gy/28 fr	97.1%	50%	—	hypopituitarism: 48%
Kopp et al. (80)	2013	37	-1.5-2mm slice -gadolinium	—	49.4 Gy/30 fr (45–52.2 Gy)	91,9%	38% (MT: 3months)	—	-hypopituitarism: 43% -vision: 5%
Lee et al. (43)	2014	64	—	—	25Gy (12–30)	100%	-Acromegaly:68.8% (MT: 24months) -Cushing: 71.4% (MT: 10 months) -Prolactinoma:50% (MT:13months)	Margin and maximum dose	-hypopituitarism: 43.5% (MT: 46months) -cranial neve paralysis: 6.3%
Liao et al. (73)	2014	34	1mm slice	5.05+-3.1	21Gy/3fr	100	—	—	—
Losa et al. (44)	2017	543	-1.5T -1mm slice -gadolinium	NFPT:1.5 (0.8-2.6) FPT:0.9 (0.5-1.7)	-NFPT: 15 (15–15) Gy, -FPT 25 (21–25) Gy	-NFPT 90.4%, -FPT 95.2%	—	marginal dose	—
Narayan et al. (29)	2018	111	yes	3,8 (0.5-19.1)	-NFPT: 15 Gy (8–40); -FPT: 17.5Gy (11.5-25)	90%	40% (MT: 23 months)	< 50 years <5cm3	-hypopituitarism: 20.4% -visual field: 9.1%, -cranial paralysis: 5.5% -hydrocephalus: 2.7% -radionecroses: 1.8%
Plitt et al. (81)	2019	53	-T1/T2/Fat sat gadolinium	6.2	46.7Gy (45-50.4) 25-28 fr	98.1%	75%	—	hypopituitarism: 1.9%
Puataweepong et al. (60)	2015	115	-1.25mm slice	SRS: 1.7; FSRT=10	-SRS: 16.8Gy (10.7-22.4Gy); -FSRT:45Gy (43-59Gy)/25fct	-SRS=93% -FSRT=95%	37.5%: (MT: SRS=16months, FSRT=20months)	—	-hypopituitarism: 19% -visual deficit:5.7%;
Scheick et al. (82)	2016	116	CT or MRI	1.9 (0,5–5,0)	45 Gy (43–55)/25fr	-NFPT: 96% -FPT: 79%	64% at 10 years	—	hypopituitarism: 26%
Sheehan et al. (83)	2011	418	—	1.9 (0,1–3)	24 Gy (9–30)	90.3%	-acromegaly: 53% (MT:29.8months) -Cushing: 54% (MT:13months) -Prolactinoma: 32% (MT:24 months) -Nelson: 22% (MT:50 months)	LC: high margin dose; HC: smaller tumor	-hypopituitarism: 24.4% -visual: 2%; -cranial nerves deficit: 1.1%;
Shrivastava et al. (84)	2019	36	Thin slice	0.5 (0,1-26.7)	25 Gy (12–40)	87.5%	-Cushing: 80% (MT:12months); -acromegaly: 42% (MT:30 months)	Margin dose	hypopituitarism: 19% (MT: 18months)
Wattson et al. (62)	2014	165	yes	1.7	Proton therapy: - SRS:20 Gy - NFRT: 50.4 Gy	98%	-All: 42% -Cushing: 54% (MT: 32months) -Nelson: 63% (MT:27 months) -Acromegaly: 26% (MT:62 months) -Prolactinoma: 22% (MT:60 months) -TSH: 33%		-hypopituitarism: 62% (MT:40 months) -seizure: 3%
Xu et al. (85)	2012	262	yes	1.6(0.1-3)	25 Gy (3–30)	89%	75%	—	hypopituitarism: 30% (MT: 30 months)
Zeiler et al. (86)	2013	86	-1mm slice -Fat Sat gadolinium	5.4(0.3-14.3)	-NFPT: 14.2Gy -FPT: 23.6 Gy	98.7%	-Acromegaly: 45% (WN: 30%); -Prolactinoma:71.4% (WM: 42.9%) -Acromegaly:100% (WM: 50%); -FSH:0%	—	-Hypopituitarism: 13.2% -visual deficit: 2.7%;

LC (local control), HC (hormonal control), NFPT (Nonfunctioning pituitary tumor), FPT (functioning pituitary tumor), SRS (stereotactic radiosurgery), FSRT (fractionated stereotactic radiosurgery), NFRT (normo fractionated radiotherapy), MT (median time), HN (hormonal normalization), WM (hormonal normalization without medication), QoL (quality of life).

In term of specific tumor subtypes, 25 articles focused on patients with NFPT, 39 on acromegaly patients, 24 on Cushing’s disease, 18 on prolactinoma, 5 clinically functioning gonadotroph PiNETs, 5 on Nelson syndrome and 3 on TSH secretion.

### Radiotherapy technique and prescription

Regarding radiotherapy techniques, the analysis revealed that normo-fractionated (NFRT) was employed in 15 articles, hypo-fractionated stereotactic irradiation (FSRT) in 7 articles, and single-dose stereotactic radiation (SRS) in 42 articles. Among these, the gamma-knife (GK) was the most used device, being employed in 38 articles, followed by linear accelerator (LINAC) in 16 articles, cyber-knife in 3 articles, and proton-therapy in one article.

For lesion delineation, 42 articles reported using MRI, while the specific imaging type was not mentioned in the remaining articles. Among the 42 articles, 22 articles mentioned the use of contrast enhancement injection, and 11 performed a Fat Sat (Fat Saturation) acquisition. The slice thickness was mentioned to in 20 articles, ranging from 0.9 mm to 3 mm. Among these articles, 7 utilized 1 mm-thick slice (**Tables 1**-**6**).

#### Volumes

Six articles described the delineation of GTV (gross tumor volume) as the “visible lesion” (12–17). In others, the delineation method involved a discussion among the neurosurgeon, radiation oncologist and physicist or it was left at the discretion of the practitioner. Nine articles described CTV (clinical target volume). The entire sellar region was included as the CTV if the lesion was not visible, in six articles (12, 18–22). In four articles, the described CTV encompassed a high-risk recurrence region, such as the cavernous sinus (12–15, 17).

The medians of the tumor volumes ranged from 0.78 to 5.41 cm^3^. Tumor volumes varied based on the subtype of PiNETs, with larger volume observed for NFPT compared to functioning pituitary tumor (FPT). Tumor volume also appears to be more limited when the fractionation is lower, with a range of 0.9 to 5.4 cm^3^ for SRS, 2.0 to 10 cm^3^ for FSRT, and 1.9 to 19 cm^3^ for NFRT. (**Tables 1**-**6**).

#### Protocol of radiotherapy

Because of the devices used to irradiate, SRS was employed in 42 articles, NFRT schedule in 15 series and FSRT in 7 studies. For the latter, the number of fractions ranged from 3 to 28. One study used a simultaneous integrated boost (SIB) (33) (**Tables 1**-**6**).

#### Dose of radiation

For NFPTs, the doses used for SRS, FSRT, and NFRT varied greatly. For SRS, the range was 12-14 Gy. As for FSRT, different schedules were published, including 17-21 Gy in 3 fractions, 22-25 Gy in 5 fractions, or 31.8 Gy in 8 fractions. In NFRT, standard doses ranged from 45 to 50.4 Gy, with 1.8 or 2 Gy per fraction.

For FPTs, the doses used for SRS, FSRT, and NFRT were 15.8 to 28.5 Gy for SRS, 21 to 30 Gy in 3 fractions for FSRT, and 45 to 56 Gy with 1.8 or 2 Gy per fraction for NFRT. In investigations involving various categories of functioning pituitary tumors, a uniform dosage regimen was applied irrespective of the specific secretion profile. Upon examining the dose ranges, they appear to exhibit comparability across distinct subtypes of secreting PiNET **Table 7**.

**Table 7:** Technic of radiotherapy and fractions used in studies.

NFPT	FPT
SRS: 12-24Gy	SRS: 15.8-28.5 Gy
FSRT:17-21Gy/3fractions; 31.3Gy/8fractions; 22-25Gy/5fractions	FSRT: 21-30Gy/3 Fractions
NFRT: 45-50.4 Gy	NFRT: 45-56 Gy

NFPT (Nonfunctioning pituitary tumor), FPT (functioning pituitary tumor), SRS (stereotactic radiosurgery), FSRT (fractionated stereotactic radiosurgery), NFRT (normo fractionated radiotherapy).

### Disease control

#### Imaging local control

Whatever the PiNET subtypes, the local control (defined as the control of the tumor growth on imaging) exhibited rates ranging from 79% to 100%.

For NFPTs, the overall local control rates ranged from 88% to 100%; for SRS, FSRT, and NFRT, ranges were 88%-100%, 88%-100%, and 91%-100%, respectively.

In cases of clinically functioning somatotroph tumors, the local control rates ranged from 85.2% to 100%, with rates of 97% for SRS, 85.5%-100% for FSRT, and 97%-100% for NFRT.

For clinically functioning corticotroph, lactotroph pituitary tumors only SRS was studied, and the results ranged from 91% to 100%, 92% to 100%, and 92.9%, respectively.

Many studies did not differentiate the outcomes based on PiNET subtypes and showed similar results. The local control rates for these studies were for SRS from 89% to 100%, for FSRTs from 98% to 100%, and for NFRTs from 79% to 100% **Table 8**.

**Table 8:** Local control rates.

	NFPT	FPT				
		GH	ACTH	Prolactin	Nelson	NS
**SRS**	88-100%	85.3-100%	91-100%	92-100%	92,9%	89-100%
**FSRT**	88-100%	97%	—	—	—	95-100%
**NFRT**	91-100%	97-100%	—	—	—	79-100%
**Proton**	—	—	—	—	—	98%

NFPT (Nonfunctioning pituitary tumor), FPT (functioning pituitary tumor), SRS (stereotactic radiosurgery), FSRT (fractionated stereotactic radiosurgery), NFRT (normo fractionated radiotherapy), growth hormone (GH), adrenocorticotropic hormone (ACTH), NS (none specified).

In a single study exclusively focusing on clinically functioning thyrotroph tumor, SRS was used in 68% of the cases, while conventional radiation therapy was used for the remaining cases. The local control rate in this study was 78%. Tumor size decreased in 26% of the cases and remained stable in 52% (23).

Gopolan et al. conducted a study to investigate the kinetics of volume changes in NFPTs treated with a GK. They observed that the mean and median time for NFPTs to decreased in volume were 28.5 months and 33 months, respectively, with a range from 6 to 99 months. For patients who experiences a relapse, the mean time for the lesion to increase in volume was 62.4 months, ranging from 20-120 months (24).

#### Predictive factors of imaging local control

Extrasellar extension was significantly associated with a lower control rate especially with cavernous sinus invasion in three studies (25–27). Smaller tumor size was associated with best results, with various thresholds, < 3 cm^3^ (27), < 3.4 cm^3^ (28), < 5 ml (24), or < 5 cm (29). Gopolan et al. found that smaller than 5 ml exhibited a faster rate of decrease compared to larger tumors. Additionally, in cases of recurrence, tumors larger than 5 ml showed a faster rate of increase in volume (p=0.003) (24). Others studies have found that a large PTV (19) and higher maximal doses were associated with better outcomes (26, 30). Marginal delivered doses was reported in 6 studies (16, 19, 22, 23), but only one specified a threshold, at 12 Gy (24). Higher control rates were achieved with a single fraction of 25 Gy compared to 20 Gy (30). Less frequently, younger patients (29), and NFPTs compared to FPTs (26) were identified as predictive factors associated with better local control results.

#### Hormonal control

Hormonal control rates were defined differently across studies, as a consequence of variations in the criteria used. Some authors considered hormonal control when patients achieved normal hormone blood levels, either with or without medication. In contrast, other authors only considered control without the use of medication. In the case of acromegaly, different biological references were utilized, such as IGF-1, urinary cortisol, or GH levels.

As a result, whatever the pathology is, the range for hormonal local control was quite broad, spanning from 10% to 94.7%.

Corticotroph FPTs hormonal control rates varied from 52% to 99%; for SRS, FSRT and NFRT, values of range were respectively 52-99%, 40% and 53.8%-73%.

For somatotroph FPTs, hormonal control rates varied from 28 to 88%; regarding SRS, FSRT and NFRT, the ranges of values were respectively 28-88%, 48-52%, and 30-74%.

For lactotroph FPTs, hormonal control rates varied from 10 to 66.7%; regarding SRS, FSRT and NFRT, the ranges of values were 32-66.7%, 10% and 50%, respectively.

For thyreotroph FPTs, the hormonal complete response was 63%.

For Nelson syndrome, hormonal control rates varied, for SRS, from 13.3% to 94.7% and for NFRT, from 64 to 75%.

For non-specified PiNETs, studies reported hormonal control rates for SRS ranged from 14.3 to 94.7% and for NFRT from 64 to 75% **Table 9**.

**Table 9:** FPT hormonal control rates.

	ACTH	GH	Prolactin	Nelson	NS
**SRS**	52-99%	28%-88%	32%-66.7%	13.3-94.7%	14.3%-94.7%
**FSRT**	40%	48-52%	10%		—
**NFRT**	53.8-73%	30%-74%	50%	64-75%	64-75%
**Proton**	54%	26%	22%	63%	—

SRS (stereotactic radiosurgery), FSRT (fractionated stereotactic radiosurgery), NFRT (normo fractionated radiotherapy) growth hormone (GH), adrenocorticotropic hormone (ACTH).

#### Predictive factors of hormonal local control

Better hormonal response was reported for patients with lower hormonal level before radiotherapy and this factor was found to be significant in 11 studies (20, 33–42). Moreover, higher marginal and maximum doses were shown to be more effective in achieving favorable outcomes in six studies (18, 20, 37, 39, 43, 44). Smaller tumors were apparently easier to control (13, 18, 21, 34). Older patients (13, 31, 45) and cavernous sinus invasion (45, 46) were associated with lower control rates.

### Side-effects

#### Pituitary function

Hypopituitarism following radiotherapy was observed in a varying percentage of irradiated patients, ranging from 3% to 58%. For non-functioning pituitary tumors, SRS reported rates ranged from 17.3% to 39%, although, for FSRT, reported rate was 3%. Among somatotroph FPTs treated with SRS, the occurrence of hypothyroidism ranged from 8.6% to 58%, whereas this rate ranged from 28% to 39% for NFRT. In case of Cushing’s disease, SRS results showed rates ranging from 10% to 32%. After lactotroph FPT treatments, 25% to 26.3% of hypothyroidism was reported with SRS, and 8.3% with NFRT. Studies reported non specifying PiNET subtypes retrieved hypopituitarism rates from 13.2% to 43.5% after SRS, 28% to 48% after FSRT and 1.9% to 26% after NFRT **Table 10A**.

**Table 10A:** Hypopituitarism rates.

	NFPT	FPT			NS
		GH	ACTH	Prolactin	
**SRS**	17.3%-39%	8.6%-58%	10%-32%	25%-26.3%	13.2%-43%
**FSRT**	3%	—	—	—	28-48%
**NFRT**	—	28%-39%	—	8.3%	1.9%-26%
**Proton**	—	—	—	—	54%

NFPT (Nonfunctioning pituitary tumor), FPT (functioning pituitary tumor), SRS (stereotactic radiosurgery), FSRT (fractionated stereotactic radiosurgery), NFRT (normo fractionated radiotherapy).

The articles do not always report the rate of pre-treatment hypopituitarism, nor the rate of hypopituitarism due to the tumor or surgery. In **Table 10B**, we list the articles that describe the development of pituitary function before and after radiotherapy, which could therefore be related to pituitary irradiation. Pre-radiotherapy hypopituitarism rates can be high, ranging from 15.4% to 61.7% (higher for all types and NFPTs, certainly associated with larger tumor sizes). Post-radiotherapy deficit rates for all axes combined increased from 17.3% to 58.3%. With similar rates between the different axes, deficit rates increased from 5.8% to 28.3% for the gonadotropic axis, from 11.5% to 29% for the thyrotropic axis, and from 10.7% to 32.5% for the adrenocortical axis. Surprisingly, one article shows a decrease in the rate of pituitary deficits in the treatment of NFPTs, with an 11% decrease in pituitary deficits (observed for SRS and NFRT techniques, but not for FSRT, which was cause of an increase in deficits).

**Table 10B:** Evolution of hypopituitarism before and after radiotherapy.

		Before RT	After RT			
		*Indifferent axes*	*Indifferent axes*	*LH/FSH*	*TSH*	*ACTH*
**All type**	Barber et al. (69)	44.3%	+28%	+16%		+10.7%
	Albano et al. (16)	61.7%	—	+16.7%	+25%	+22.7%
	Cohen I. et al. (70)	40%	+58.3%	+28.3%	+26.7%	+18.3%
**GH**	Bostrom et al. (15)	20.7%	+46.4%	—	—	—
	Gonzales-V. et al. (36)	35%	+41%	+20%	+29%	%32.5%
	Wu et al. (42)	36%	+17.3% (-11.1%)	—	—	—
**ACTH**	Balossier et al. (71)	15.4%	+26.9%	+5.8%	+11.5%	+15.3%
**Prolactin**	Cohen I. et al. (25)	31.6%	+25%	—	—	—
**NFPT**	Wilson et al. (50)	65%	-11%	—	—	—

Radiotherapy (RT), LH/FSH (luteinizing hormone/follicle stimulating hormone), TSH (thyroid stimulating hormone), ACTH (adrenocorticotrophin hormone), GH (growth hormone), NFPT (nonfunctioning pituitary tumor).

A larger volume was found to be associated with a higher frequency of pituitary deficits (16, 55, 58), particularly when the whole sellar was targeted. Among the affected pituitary axes, the thyrotropic, corticotropic, and gonadotropic axes were found to be the most frequent altered functions (20, 32, 36, 47).

In their study, Oh et al. specifically investigated hypopituitarism after GK treatment in patients with NFPTs (47). Among 76 patients who received the treatment, 23.5% developed *de novo* pituitary deficit, with 7.5% of patients requiring medication to manage the condition. They identified significant predictor factors associated with the development of hypopituitarism, including larger PTV, and a shorter distance between the tumor and the pituitary stalk. The mean radiation dose did not show a significant difference. However, the study established dose thresholds of 7.56 Gy for the mean dose and 12.3 Gy for the maximum dose to the stalk. Exceeding these dose thresholds was associated with a sensitivity of 76.9% and a specificity of 69.2% in predicting the development of hypothyroidism.

#### Visual side effects

There were only few visual side-effects, there incidence ranged from 0% to 9.5%. these visual complications were not always specified or graded, leading from decrease of vision acuity, sometimes resulting to blindness. NFPTs had 0-5.5% of visual side effects with SRS; 0% with FSRT; and 0-3.8% in NFRT.

Regarding somatotroph FPTs, the reported of treatment-related visual complications rates were, for SRS, from 0% to 9.5%; for FSRT, 2.85%, and for NFRT from 0% to 1%. In the case of corticotroph FPTs, only SRS was documented, and the reported treatment-related visual complications rates ranged from 0% to 7.7%. For studies that did not specify the subtype of PiNETs, the reported treatment-related visual complications rates were, for SRS, from 0% to 6.3%; for FSRT, 0%; and for NFRT, the response rate ranged from 0% to 1.5% **Table 11A**.

**Table 11A:** Visual side effects rates.

	NFPT	FPT		NS
		Acromegaly	Cushing	
**SRS**	0%-5.5%	0%-9.5%	0%-7.7%	0%-6.3%
**FSRT**	0%	2.85%	—	0%
**NFRT**	0%-3.8%	0%-1%	—	0%-1.5%

NFPT (Nonfunctioning pituitary tumor), FPT (functioning pituitary tumor), SRS (stereotactic radiosurgery), FSRT (fractionated stereotactic radiosurgery), NFRT (normo fractionated radiotherapy).

In **Table 11B**, we have summarized the articles describing the pre-treatment rate of visual impairment and its evolution after radiotherapy, in particular the cases of visual improvement and deterioration. Before radiotherapy, the rate of visual impairment varied between 0% and 55.9%, with higher rates for PiNETs of all types and for NFPTs (46.7% to 55.9%). After radiotherapy, visual impairment worsened in 0% to 7.7% of patients and improved in 5.5% to 36% of patients.

**Table 11B:** Evolution of visual disorders before and after radiotherapy.

		Before RT	After RT	
			*Worse*	*Improved*
**All type**	Albano et al. (16)	55,5%	+2,3%	+ 36%
	Barber et al. (69)	46,7%	+1,5%	+ 15,7%
**GH**	Balossier et al. (33)	11,9%	+0%	—
	Bostrom et al. (15)	0%	+2,9%	—
	Wu et al. (42)	27,1%	+ 4,9%	+6,1%
**ACTH**	Balossier et al. (71)	7,7%	+7,7% (transients)	—
**NFPT (peri optical tumors)**	Hata et al. (72)	53%	+6,6%	+34,3%
	Liao et al. (73)	55,9%	+0%	+5,5%

RT (Radiotherapy), ACTH (adrenocorticotrophin hormone), GH (growth hormone), NFPT (nonfunctioning pituitary tumor).

For NFPTs, the two studies reported, provided descriptions of the visual effects associated with tumors clos to optical pathways. The results substantiate the efficacy of radiotherapy, with a higher incidence of visual amelioration (ranging from +5.5% to +34.3%) compared to instances of deterioration (0% to 6.6%).

#### Neurocognitive and neurovascular side-effects

Neurocognitive effects were studied specifically by Lecumberri et al. Patients were treated with GK SRS. Authors showed significant worst performance on Barcelona’s story recall test (p = 0.001), arithmetic’s problems (p = 0.03), perseverative answers and errors of Wisconsin card sorting test (p = 0.001) (48). However, Tooze et al. reported no significant difference in intelligence memory or executive function for patients treated with GK SRS (49). Wilson et al. reported only 1% loss of memory in irradiated patients with 2% for patient treated with SRS, 0% with FSRT and 4% with NFRT (50). Crouzeix et al. Crouzeix et al. compared neurocognitive alterations and quality of life in patients with NFPT who received radiotherapy and those who did not. Cognitive functions were found to be mildly altered in patients with NFPT but radiotherapy did not obviously contribute to such dysfunction. The mental dimension of QoL was moderately affected in patients who received radiotherapy while its physical dimension showed no deterioration (51).

Some articles reviewed the neurovascular effects, from one case to 10.5% of treated patients (13, 52, 53).

## Discussion

This review analyzed a substantial pool of retrospective studies, collectively demonstrating the efficacy of radiation therapy in treating PiNETs (local control range: 79%-100%; hormonal control range: 10-94,7%). These studies had considerable median follow-up periods ranging from 33 to 198 months. The lack of prospective studies can be attributed to the low incidence of these tumor and the necessity of long-term follow-up to validate the treatment outcomes.

MRI is widely regarded as the gold standard for evaluating the pituitary region and devising the planning treatment. Contrast-enhanced fat-suppressed T1-weighted (Fat sat) sequences appear to be beneficial after surgical treatment as they reduce the obscuring effects caused by post-surgical modifications and augment the contrast between the tumor and adjacent structures (54). The slices should be as thin as possible considering that microtumors can be smaller than 5 mm. Additionally, a coronal acquisition could aid in delineating tumors that have a superior-inferior extension (55).

Delineation guidelines for PiNETs are often lacking in detail. The process is not precisely described and can vary depending on individual practices, which introduces a significant bias when comparing treatment results without a clear understanding of what is being clearly treated.

However, there are recent recommendations provided by the ESTRO (European Society for Radiotherapy and Oncology) (56). According to these guidelines, the gross tumor volume (GTV) is defined as the visible tumor on thin-slice MRI (specifically, on T1-weighted enhanced images using gadolinium). The clinical target volume (CTV) is not mandatory, but if there are criteria indicating invasiveness or aggressiveness, a margin of 2-3 mm can be applied. In stereotactic conditions, the planning target volume (PTV) margins range from 1-3 mm, while in conventional radiotherapy, a margin of 5 mm is used. These guidelines aim to provide more standardized and consistent approaches to delineating the pituitary tumors, taking into account the tumor visibility on MRI and various treatment conditions (56). The volume required to be irradiated is currently questioned depending on the type of PiNET. For NFPTs only the lesion could be targeted, while for FPT, the entire sella could be treated to minimize the risk of missing active secreting cells spread throughout the sella. Three series of studies have described irradiating the entire sellar region, suggesting that this approach may be appropriate in certain cases (18, 41, 43). Lee et al. conducted a study involving 64 patients with FPTs who experienced biological relapse post-surgery, with no visible tumor or infiltrative tumor prior to surgery. The patients were administered a median single dose of 25 Gy to the whole sellar. The achieved control rates were not different to the other published series, with 68.8% for acromegaly, 71.4% for Cushing’s disease, and 50% for prolactinoma. The authors did not report a dramatic rate of possibly radiation-induced onset of hypothyroidism with a rate of 43.5%. Notably, predictive factors for hypothyroidism and control rates were dose margin and maximum dose (43).

Shepard et al. conducted a study comparing whole sellar irradiation versus tumor-targeted irradiation in patients with Cushing’s disease, using stereotactic radiosurgery (SRS) with a mean margin dose of 22.4 Gy at a median isodose of 50% (30-60%). The study found no significant difference in terms of efficacy between the two approaches, as well as no significant difference in the development of induced endocrine deficits (18). Taylor et al. conducted a similar comparison study involving 128 patients with Cushing’s disease with comparable results in terms of disease control rates. However, they observed a potentially higher incidence of mortality and visual deficits in the whole sellar group, although this difference did not reach statistical significance (41).

Radiotherapy is generally considered a second- or third-line treatment option for PiNETs after surgery or medical therapy (4). While the side-effects of surgery and medication cannot be directly compared to radiation-induced effects, each treatment option has shown acceptable results and similar rates of complications or side effects. In a 2018 review, Alzhrani et al. studied the complications of transsphenoidal surgery for PiNETs. He described few pituitary deficits ranging from 3% to 6.1%, and a rate of 3-19% of central hyponatremia, 0.3%-3.5% epistaxis, 1.1% carotid pseudoaneurysm, 5.8% of hydrocephalus and 29.6% sino-nasal disorders (57).

Indeed, two sets of studies have investigated the use of radiotherapy as a first-line treatment. Wu et al. conducted a comparing study of 75 patients with acromegaly retrospectively recorded. Among them, half received stereotactic radiosurgery (SRS) as the first-line treatment, while the other half underwent radiation postoperatively. The indication of primary SRS was for patients unable to tolerate surgical risks or unwilling to undergo surgery. The indication for post operative SRS was for those who suffered recurrent or residual lesion after surgical removal. The postoperative group exhibited a statistically younger mean age (p = 0.028), a higher proportion of female patients (p = 0.010), larger tumor volume (p = 0.042), and a greater number of cases with Knosp grade ≧ 3 (p > 0.001). The hormone levels before SRS did not significantly differs between the two groups. In the postoperative SRS group, the median duration from surgery to SRS was 7 months (range: 3− 97 months). The median maximum dose and margin dose of the postoperative GKS group was significantly lower than that in the primary group (p = 0.014, p = 0.008). Biochemical recurrence rate was 2.63% in the primary group and 5.41% in the postoperative group (p = 0.981). Durable endocrine remission rate was 21% in the primary group and 21.6% in the postoperative group (p = 0.831). Only the base nadir GH level after treatment was identified as a predictor of durable biochemical remission (HR = 0.829,95% CI:0.704− 0.976; p = 0.025). So the study found no significant difference in endocrine remission, biochemical recurrence, imaging outcomes, or complications between the two groups (42). This study demonstrates the effectiveness of radiation therapy as a first-line treatment compared to a group considered to have experienced surgical failure due to incomplete removal of the lesion or relapse on it. However, these findings do not preclude the need for a direct comparison between up-front irradiation and complete removal of the lesion.

Yu et al. studied the outcomes of 81 patients with NFPT treated with a single session of GK as their first-line treatment. Forty-eight patients (59.3%) presented a lesion with suprasellar extension. The results found that the complete response (CR) rate was 88.9%, and tumor treatment failures were confirmed in nine patients (11.1%), indicating a high rate of tumor control. Fourteen patients (17.3%) developed hypopituitarism, including hypogonadism (n = 7), hypothyroidism (n = 8), and hypercortisolism (n = 7). This rates remained moderate compared to other studies (32).

These findings suggest that radiation therapy can be considered as a viable first-line treatment option for PiNETs, with outcomes comparable to those of surgery or medical therapy. However, individual patient factors and tumor characteristics should be considered when making treatment decisions, and a multidisciplinary approach is often necessary to determine the most appropriate treatment strategy. However, these factors still need to be clearly defined.

Regarding irradiation techniques, monofractionated irradiation is overrepresented in the published article. Several reasons can explain this observation: i/tumors mostly remain as small tumors confined to the sellar region, allowing for high doses per fraction; ii/The limited ability of PiNET cells to spread out permits the use of smaller or no margins around the GTV, which is favorable for high doses per fraction; iii/patients were often initially referred to neurosurgeons for surgery, but they frequently have access to Gamma Knife (GK) treatment, providing an opportunity for both treatments; iv/In terms of quality of life, monofractionated treatment is less restrictive for patients; v/finally, in terms of cost, monofractionated irradiation is often less expensive than fractionated irradiation. FSRT was less commonly used in NFPTs, possibly because the lesions tend to be smaller, and the need for fractionation to protect organs at risk is less frequent. NFRT appears to be less likely to be associated with visual deficits, but the conclusions are limited due to a lower number of studies and smaller patient populations, particularly in the case of stereotactic and FPTs. In **Table 8**, the local control rates ranged from 85% to 100%. based on radiation technique. No technique appears to be superior, and all PiNETs subtypes exhibit excellent response rates.

In terms of hormonal control rates, results remained good but with wide and overlapping intervals (**Table 9**), whatever the technique used. However, when examining specific subtypes such as prolactinomas, the intervals for hormonal control rates tend to be lower (ranging from 10% to 66%), compared to acromegaly (26% to 88%) and Cushing’s disease (40% to 99%).

However, comparison between techniques is not possible in this review. There isn’t much data comparing the different radiotherapy techniques, but IMRT/VMAT irradiation seems to provide better coverage and protection of adjacent structures, although it exposes them to more low doses (58). This could be theoretically improved by proton therapy, but no high-quality data currently favors one technique over another (10, 59).

Consequently, the choice of technique should not solely depend on the tumors subtype but also on the need to protect sensitive structures near the treated area. In studies where the tumor is in close to the optic nerves or optic chiasm, fractionation is often employed to minimize the risk to these at risk structures (16, 60). Tumor size is also a factor, as larger tumors tend to necessitate fractionation in treatment planning (15, 27, 50). NFPTs were considered to be more radioresistant, so higher doses were often administered. However, no radiobiological studies investigating radiosensitivity *in vitro* could be found in the literature to confirm this hypothesis.

The dose and treatment planning of radiotherapy for PiNETs should take into consideration various predictive factors. Factors such as tumor size, invasiveness, hormonal levels, and patient age can guide the decision-making process for selecting the appropriate margin and maximum dose. The aim is to balance tumor control with minimizing the risk of radiotherapy-induced side effects (10).

Since PiNETs are often considered as benign tumors, it is crucial to choose a treatment option with the least side effects and good tolerability. One of the major concerns of radiation treatment is the potential development of hypopituitarism, which refers to a deficiency in pituitary hormone production (61). **Table 10A** demonstrates the variability in hypopituitarism rates, with overlapping intervals and rates ranging from 1.9% to 58%. Interestingly, even proton therapy, which is often considered to have better sparing of surrounding tissues, exhibits a relatively high rate of pituitary deficits at 54% (62).

And **Table 10B** describes the evolution of hypopituitarism rates before and after radiotherapy. This shows that before radiotherapy, pituitary function is often impaired in 15.4-65% of treated patients, especially in cases of NFPT (in cause the compressive nature of non-secreting macrotumors). The rate of new cases of hypopituitarism related to radiotherapy is moderate, with a wide range from +17.3% to +58.3%, and affects equally the corticotropic and thyrotropic gonadotropic axes.

The predictive factors for radiotherapy-induced side effects in PiNETs treatment include a larger tumor volume and a shorter distance between the tumor and the pituitary stalk. However, the article by Deng et al. comparing radiotherapy versus no radiotherapy after surgery found no significant differences between the groups in terms of hormone deficiency at presentation, at 3 months postoperatively, or at the last follow-up. This suggests that the addition of radiotherapy did not significantly impact hormone deficiency rates in this study (27).

In the study by Wilson et al., which treated 176 patients with NFPTs using different radiotherapy techniques (SRS, FSRT, CRT), the risk of new pituitary deficits was assessed by comparing pre- and post-treatment hormonal replacement. The results showed that for SRS and FSRT, there was an improvement in preexisting hypopituitarism in 5 patients (10%), but a new deficit was observed in 4 (7%) new patients and requiring hormonal replacement after treatment, while for CRT 17 patients (32%) experienced a correction of their hypopituitarism. This suggests that conventional radiotherapy (CRT) had a higher rate of sparing pituitary function compared to SRS and FSRT (50).

Indeed, visual side effects are less frequent in pituitary tumors radiotherapy, and the intervals in **Table 11A** overlap, indicating similar rates among different techniques. However, if we look at the upper intervals, SRS seems induce higher rates of visual deficits (ranging from 5.5% to 9.4%) compared to FSRT (2.85%) and CRT (1% to 3.8%). Although the lower intervals are all at 0%, these findings suggest that SRS may have a slightly higher risk of visual deficits, emphasizing the importance of fractionating the dose when the planning target volume (PTV) is near the optic pathways. In **Table 11B**, we can see that there are higher rates of improvement in visual function (5.5%-34.5%) than deterioration (0-7.7%) after radiotherapy, so the risk-benefit ratio is in favor of radiotherapy from this point of view.

Other side effects, such as neurocognitive effects, stroke, seizure, radionecroses, or radio-induced brain tumors are occasionally described in some studies. For example, Sattler et al. compared the incidence of stroke in patients treated with surgery alone versus surgery plus radiotherapy (mostly NFRT with a dose of 45 Gy) with a median follow-up of 14 years. The study found that postoperative radiotherapy in PiNET was not associated with an increased incidence or causative mechanism of stroke compared to patients treated with surgery alone (63). A large size retrospective cohort involving 4292 patients treated with radiotherapy for pituitary adenomas or craniopharyngiomas found an association between radiotherapy and an increased risk of second brain tumors. The rate ratio for irradiated patients was 2.18 (95% CI 1.31–3.62, p < 0.05) (8).

The results of local and hormonal controls must be correlated with their real impact on patients’ lives, particularly in terms of improving clinical symptoms and quality of life.

In the case of a large tumor, whether secretory or not, the aim is to control the size of the tumor in order to avoid visual symptoms, damage to the cranial nerves, headaches or intracranial hypertension syndrome. In this context, controlling or even reducing the size of the tumor may alone lead to an improvement in symptoms. However, there is also a risk of radiotherapy induced neuropathy of cranial nerves, although radiation doses generally remain below the usual constraints (optic tracts: NFRT: <54Gy, SRS: <8 Gy, SFRT: 13,7Gy/3Fractions to 29,6Gy/8fractions) (64). As the **Table 11B** shows, the benefit-risk balance favors radiotherapy, which appears to result in a better improvement in visual function compared with post-radiotherapy visual disorders.

For clinically functioning pituitary tumors, particularly small recurrent tumors or the ones that are difficult to localize, the therapeutic approach differs. There are several lines of medication available to control hormone secretion, and radiotherapy is indicated later if medical treatments fail, are poorly tolerated or are contraindicated. In the case of Cushing’s disease in particular, drug treatments are more complex to manage, and tolerance is uncertain. In addition, the hypercortisolism induced by ACTH hypersecretion carries a significant risk of morbidity, particularly cardiovascular morbidity, hence the importance of hormonal control. Radiotherapy appears to be a wise choice after failure of drug or surgical treatment. On the other hand, in the case of hyperprolactinemia, hypersecretion is generally not very symptomatic and can be easily controlled by medication. In this case, radiotherapy is aimed more at reducing the volume of the tumor (65–67).

With regard to the assessment of radio-induced pituitary deficits, not all articles specify which specific hormonal axes are affected, as not all deficits have the same impact on the patient. A corticotropic or thyrotropic deficiency has a real daily impact, requiring strict supplementation and presenting vital risks, particularly in the case of acute adrenal deficiency. Prolactin deficiency, on the other hand, has no major impact on the patient, and the effects of growth hormone (GH) deficiency remain debated in adulthood. For FSH/LH deficiency, supplementation is generally straightforward (67, 68).

In **Table 10B**, when focusing only on hormonal axes that have an impact on patients, we observe an incidence ranging from 5.8% to 28.8% for LH/FSH, 10.7% to 32.5% for ACTH, and 11.5% to 29% for TSH. This could potentially affect up to one-third of patients, and thus, this data should always be weighed against the anticipated benefits for the patient. In fact, if the objective is to reduce the treatment burden for a patient with hypersecretion, radiotherapy might potentially lead to a reduction in hypersecretion. However, it comes with the risk of inducing a new deficiency that would require medication, which could be more or less demanding.

Overall, these findings indicate that the choice of radiotherapy technique may influence the risk of new pituitary deficits. However, it’s important to note that individual patient factors and tumor characteristics can also play a role in determining the impact of radiotherapy on hormone deficiency. Therefore, treatment decisions should be made on a case-by-case basis, considering the specific characteristics and needs of each patient.

## Conclusion

Radiation therapy is an effective treatment option for both NFPT and FPT, with high rates of local control and good hormonal control. However, it is important to note that the review is skewed towards SRS, and there is a lack of studies directly comparing SRS, FSRT, and CRT. The major side effect of radiotherapy is hypopituitarism, which can affect up to half of the patients. Prospective studies with larger sample sizes and direct comparisons between different techniques are needed to obtain a higher level of evidence and further elucidate the optimal approach for PiNETs radiotherapy.
